# Regulation of Transcription Factor E2-2 in Human Plasmacytoid Dendritic Cells by Monocyte-Derived TNFα

**DOI:** 10.3390/v12020162

**Published:** 2020-01-31

**Authors:** Hannah K. Dewald, Harry J. Hurley, Patricia Fitzgerald-Bocarsly

**Affiliations:** 1 Rutgers School of Graduate Studies, Newark, NJ 07103, USA; hkd17@gsbs.rutgers.edu (H.K.D.); hjh42@njms.rutgers.edu (H.J.H.); 2 Department of Pathology, Immunology, and Laboratory Medicine, Rutgers New Jersey Medical School, Newark, NJ 07103, USA

**Keywords:** plasmacytoid dendritic cell, E2-2, TCF4, transcriptional regulation

## Abstract

Plasmacytoid dendritic cells (pDCs) are innate immune cells and potent producers of interferon alpha (IFNα). Regulation of pDCs is crucial for prevention of aberrant IFN production. Transcription factor E2-2 (TCF4) regulates pDC development and function, but mechanisms of E2-2 control have not been investigated. We used freshly-isolated human peripheral blood mononuclear cells stimulated with toll-like receptor 7, 9, and 4 agonists to determine which factors regulate E2-2. After activation, pDCs decreased E2-2 expression. E2-2 downregulation occurred during the upregulation of costimulatory markers, after maximal IFN production. In congruence with previous reports in mice, we found that primary human pDCs that maintained high E2-2 levels produced more IFN, and had less expression of costimulatory markers. Stimulation of purified pDCs did not lead to E2-2 downregulation; therefore, we investigated if cytokine signaling regulates E2-2 expression. We found that tumor necrosis factor alpha (TNFα) produced by monocytes caused decreased E2-2 expression. All together, we established that primary human pDCs decrease E2-2 in response to TNFα and E2-2 low pDCs produce less IFN but exhibit more costimulatory molecules. Altered expression of E2-2 may represent a mechanism to attenuate IFN production and increase activation of the adaptive immune compartment.

## 1. Introduction

Identified as the primary type 1 interferon producing cells, plasmacytoid dendritic cells (pDCs) have garnered interest because of their capacity to produce up to 1000-fold more interferon alpha (IFNα) than any other cell [1,2,3]. This unique ability gives pDCs a critical role in protection against pathogens [4]. pDCs are innate immune cells that sense viral ssRNA and DNA through toll-like receptors (TLR) 7 and -9, respectively. Stimulation through each of these TLRs converge on the same downstream pathway, ultimately leading to the phosphorylation and translocation of interferon regulatory factor 7 (IRF7) and transcription of Type I IFNs [5,6]. pDC activation is not limited to TLR7 and -9 —they also express functional TLR4 [7]. Previous work in our lab found that pDCs respond to lipopolysaccharide (LPS) through TLR4 by upreg ulating IRF7 and TLR4, ultimately priming pDCs to produce more IFN in response to a secondary viral signal [7].

Development of pDCs begins in the bone marrow, where these cells originate from a common dendritic cell (DC) progenitor and culminates with migration and maturation in the blood and tissue [2,8]. The fate of the DC progenitor is dependent on the balance of key developmental factors, including ID2, IKZF1, NOTCH1, SPIB, and TCF4 [9,10,11,12,13]. In humans, myeloid dendritic cells (mDCs) diverge from pDCs at the progenitor stage and mature into one of five different mDC populations currently identified [8,14]. pDC arise in the presence of FLt3-ligand and IL-3, which activate STAT3. STAT3, in turn promotes the expression of transcription factor E2-2 (TCF4) [11]. E2-2 is required for pDC development [10,11,15,16] and promotes canonical pDC phenotype and function [17].

E2-2 is a basic helix-loop-helix transcription factor that binds the E-box sequence “CANNTG.” Binding of E2-2 can either activate or repress target genes [18]. E2-2 is highly expressed in pDCs and at low levels in mDCs and B cells. In pDCs, E2-2 promotes several archetypal pDC genes, such as SPIB, ILT7 and TLR7/9, and represses mDC-associated genes [4,10,17]. In mice, whole system E2-2 knockout ablates all pDC development [10,16], while inducible knockout of E2-2 in peripheral pDCs causes a conversion to an mDC-like cell with altered phenotype and function [15]. This is marked by expression of mDC markers CD11c and CD8 and a complementary loss of pDC markers, such as CIITA. Change in phenotype is accompanied by a significant reduction in the capacity for Type 1 IFN production and increased ability to stimulate T cells [15].

Studies of pDCs have indicated that mature pDCs alter E2-2 expression in response to a variety of stimuli [16,19]. In vitro, human pDC cell lines that downregulate E2-2 exhibit a similar phenotype and function as described in murine E2-2 knockout pDCs [17]. However, human pDC cell lines do not completely recapitulate the transcriptional and functional profile of primary pDCs [20]. Particularly, they are poor producers of type I IFN and can produce a significant level of interleukin 12 [21,22,23]. mDCs that display both pDC and mDC characteristics and rely on E2-2 for development have been identified in mice, and human tonsils and blood [8,14,24,25,26]. The presence of these cells indicates that there may be a population of DCs that are actually pDCs that downregulated E2-2 in the periphery. In the context of chronic viral infection, we observed that pDCs from HIV-infected donors have decreased expression of E2-2 [27]. pDCs from people living with HIV are both numerically and functionally deficient [reviewed in [28]. Together, these data indicate that E2-2 expression can be altered in result of physiological conditions which may lead to the development of a DC with pDC and mDC characteristics.

Until now, the upstream processes that contribute to E2-2 regulation in the context of human pDC activation have remained unstudied. In this investigation, we determined the temporal regulation of E2-2 in response to TLR 7, 9, and 4 stimulation and found that downregulation occurs after 18–24 h after the start of stimulation. In congruence with previous literature, we confirmed that pDCs that decrease E2-2 expression produce less IFN and express costimulatory markers [15]. We identified a mechanism by which TNFα produced by monocytes attenuates E2-2 expression in pDCs. pDC dysfunction is attributed to the pathogenesis of several autoimmune diseases [29,30] and has been observed in chronic HIV infection [28,31,32]. We provide novel insights in the regulation of pDCs which may be further used to promote or attenuate pDC function during disease progression.

## 2. Materials and Methods

### 2.1. Preparation of PBMC

This study was approved by the Institutional Review Board of New Jersey Medical School, ID Pro0119980237, approved November 14, 2019. Whole blood was collected from healthy, consenting adults in heparinized collection tubes. Peripheral blood mononuclear cells (PBMCs) were isolated using Lymphocyte Separation Medium (Corning, Manassas, VA, USA) via density centrifugation following the manufacturer’s protocol. PBMCs were cultured in RPMI 1640 (VWR Life Science, Radnor, PA, USA) with l-glutamine (Corning) supplemented with 10% heat-inactivated fetal bovine serum (Gibco, Gaithersburg, MD, USA), 100 U/mL penicillin, 100 mg/mL streptomycin, 100 mg/mL gentamicin, and 5 mM HEPES (Sigma Aldrich, St. Louis, MO, USA). Cell number was obtained using the Cellometer Auto 2000 (Nexcelom, Lawrence, MA, USA).

### 2.2. pDC Enrichment & Monocyte Depletion

pDCs were enriched from PBMC, prepared as described above, by negative selection using the human plasmacytoid dendritic cell isolation kit-II (Miltenyi Biotec, Bergisch Gladbach, Germany) according to the manufacturer’s instructions. Purity of resulting pDC population was assessed via flow cytometry as the proportion of BDCA2 CD123 double-positive cells, with purities ranging from 90–95%. To confirm that negative selection excluded any contaminating mDC populations, purified pDCs were stained for CD11c, BDCA3, AXL, and Siglec6, which are known markers of human mDC [14]. Monocyte depletion was achieved using Miltenyi CD14 Microbeads according to the manufacturer’s protocol. Depletion was verified by identifying CD14+ cells in control or monocyte-depleted PBMCs via flow cytometry.

### 2.3. Viruses

Herpes simplex virus type 1 (HSV) strain 2931 was originally obtained from Dr. C. Lopez, then at the Sloan-Kettering Institute (New York, NY, USA). HSV stocks were expanded in VERO cells (American Type Culture Collection, Manassas, VA, USA) and titered by a plaque assay on VERO cells. Influenza A virus (IAV) strain PR/8/34 propagated in specific-pathogen free eggs was purchased from Charles River Laboratory (SPAFAS, N. Franklin, CT, USA). HIV-MN strain was obtained from the AIDS and Cancer Virus Program (National Cancer Institute at the National Institutes of Health). All viruses were stored at −80 °C and thawed at room temperature before use.

### 2.4. Antibodies

PE/Cy7 anti-CD123 (6H6), allophycocyanin anti-CD123 (6H6), BV510 anti-CD123 (6H6), BV421 anti-BDCA2 (201A), PerCP Cy5.5 anti-BDCA2 (201A), allophycocyanin anti-CD3 (HIT3a), allophycocyanin/Cy7 anti-CD11c (Bu15), AF700 anti-CD14 (HCD14), PE/Cy7 anti-CD80 (2D10), PerCP Cy5.5 anti-CD86 (IT2.2), were purchased from BioLegend (San Diego, CA, USA). PE anti-IFNα (LT27:295) was purchased from Miltenyi Biotec (Bergisch Gladbach, Germany). PE anti-TLR4 (HTA125) was purchased from eBioscience (San Diego, CA, USA). PerCP anti-AXL (108724) and AF594 anti-Siglec 6 (767329) were purchased from R&D Systems (Minneapolis, MN, USA). Unconjugated monoclonal anti-TCF4 (NCI-R159-6) was purchased from Abcam (Cambridge, MA, USA).

### 2.5. Flow Cytometry

PBMCs were surface stained and fixed with 2% paraformaldehyde. Anti-TCF4 antibody was labeled with the AF488 Zenon Kit (ThermoFisher, Waltham, MA, USA) following the manufacturer’s protocol. To stain for TCF-4, PBMCs and enriched pDCs were permeabilized using the previously published protocol [33]. Briefly, after surface staining, cells were fixed with 2% paraformaldehyde for 10 min and washed. After washing, 1 mL 80% methanol was slowly added to each sample. Samples were stored at −20 degrees overnight to allow for permeabilization. Cells were then washed and stained for E2-2. For detection of IFNα, PBMCs were surface stained, fixed and permeabilized as described above or permeabilized using Triton X-100 (Sigma Aldrich). Samples were acquired at 300,000 events using an LSR-II or Fortessa X-20 (BD Biosciences, Franklin Lakes, NJ, USA). Flow cytometric analysis was performed with FlowJo software (FlowJo, LLC Ashland, OR, USA).

### 2.6. Cell Culture

PBMCs were cultured in 5 mL round-bottom polystyrene tubes at a concentration of 2 × 10^6^ cells/mL in 10% FBS RPMI at 37 °C with 5% CO_2_. 1 mL of cell culture was used per sample. For analysis of purified pDC, 100,000 pDC in 100 µL of 10% FBS RPMI per sample were cultured in a round-bottom 96-well plate. PBMC were stimulated with a multiplicity of infection (MOI) of 1 of HSV-1 and IAV and purified pDCs were stimulated with an MOI of 10 (which yields a similar concentration of virus volume:volume as in the PBMCs). PBMCs were stimulated with 500 ng p24 equivalents/mL of HIV-MN. 200 ng/mL of ultrapurified LPS (Invivogen, San Diego, CA, USA) and 5 ug/mL CpGA (ODN 2336) and CpGB (ODN 2006) (Invivogen) were used to stimulate both PBMCs and enriched pDCs. 10 μM R848 (Resiquimod) was used to stimulate PBMCs (Invivogen). Supernatants were collected from PBMC cultures stimulated with LPS and kept at −20 °C until use. pDCs and PBMCs stimulated with supernatant were cultured in media from autologous PBMCs. For inhibition of LPS binding by polymyxin B, supernatants were pre-treated with 1 μg/mL of polymyxin B (Invivogen) for a half hour before use. For cytokine stimulation, PBMCs were cultured with 10, 100, 1000, 5000, or 10,000 IU IFNα2b or 0.2, 1, 10 ng/mL of IL-10 or TNFα (PeproTech, Rocky Hill, NJ, USA).

### 2.7. qRT-PCR

Total RNA was isolated from PBMCs with the RNeasy Micro kit (Qiagen, Germantown, MD, USA) according to the manufacturer’s protocol. Purity and concentration of RNA were measured with the nanodrop DeNovix FX11+ spectrophotometer/fluorometer (Wilmington, DE, USA). RNA was reverse transcribed to cDNA using random hexamers (Invivogen) and MuLV reverse transcriptase (Applied Biosystems, Foster City, CA, USA). cDNA was amplified via qPCR using a QuantStudio 3 to quantify mRNA expression in pDC relative to β-actin using the following Taqman gene-specific probe-based assays (Thermofisher): ACTB (Hs99999903_m1) and TCF4 (Hs00162613).

### 2.8. Enzyme-Linked Immunosorbent Assay

The presence of TNFα in supernatants was determined by enzyme-linked immunosorbent assays (ELISA). The Human TNF-alpha DuoSet ELISA kit from R&D Systems was used following the manufacturer’s protocol.

### 2.9. Statistical Analysis

Statistical tests were performed as repeated measures comparisons. Paired t-tests were used to calculate significant differences between two groups, rmANOVA with Dunnett’s post hoc test to calculate significant differences between three or more groups, and two-way rmANOVA with Dunnett’s post hoc test to calculate significant differences in experimental designs with multiple factors. All residuals were found to be normally distributed according to the Shapiro-Wilk test, and sphericity was assumed when applicable. Statistical tests were performed in GraphPad Prism 7 (San Diego, CA, USA). Data are presented as means ± SEM. *p* values < 0.05 were considered significant. * *p* < 0.05, ** *p* < 0.01, *** *p* < 0.001, **** *p* < 0.0001.

## 3. Results

### 3.1. High E2-2 Expression Is Distinctive to pDCs and Is Downregulated after Stimulation

**Figure 1:**
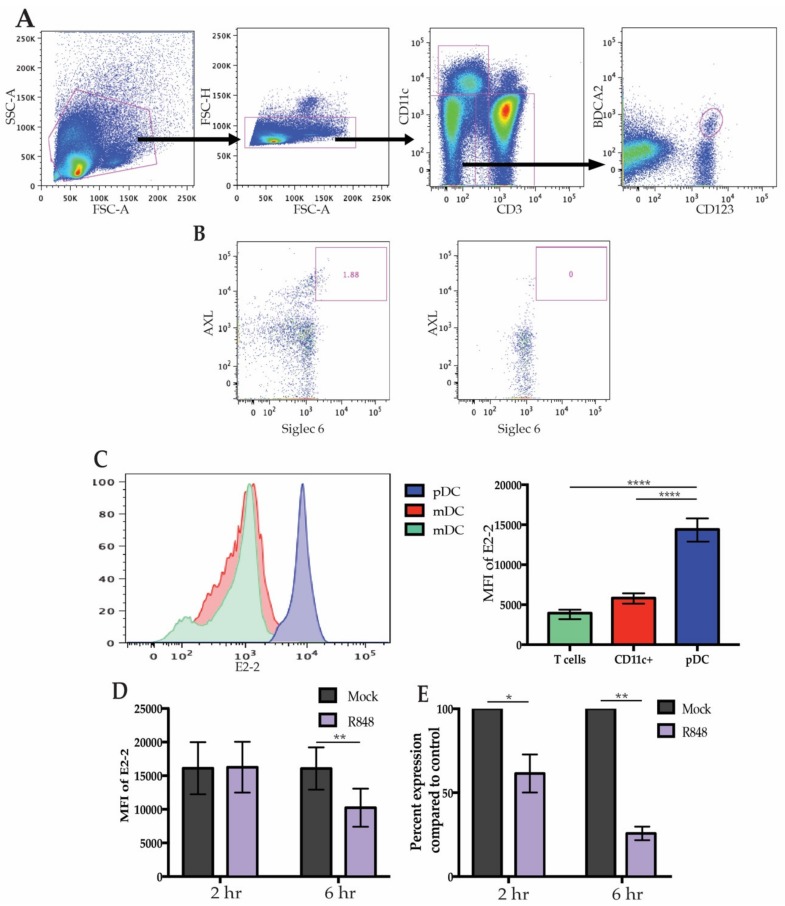
E2-2 expression in plasmacytoid dendritic cells (pDCs). (**A**) Gating strategy to identify pDCs from human peripheral blood mononuclear cells ( PBMCs). (**B**) Gating to determine removal of AXL+ Siglec 6+ DCs after negative selection for pDCs. (**C**) E2-2 expression in CD3+ and CD11c+ cells compared to pDCs as determine by flow cytometry. Representative histogram on the left, quantified mean fluorescent intensity (MFI) on the right. *n* = 11 independent experiments. (**D**) PBMCs were stimulated with 10 µM R848 for up to 6 h and E2-2 expression was measured via flow cytometry. *n* = 8 independent experiments. (**E**) mRNA expression from PBMCs measured by qRT-PCR after 6 h R848 stimulation. *n* = 3 independent experiments. Data are presented as means ± SEM. *p* values < 0.05 were considered significant. * *p* < 0.05, ** *p* < 0.01, *** *p* < 0.001, **** *p* < 0.0001.

It has previously been established that high expression of E2-2 is specific to unstimulated pDCs and there has been brief evidence that expression may be altered after activation. During chronic viral infection in both mice and humans, pDCs express significantly less E2-2 compared to healthy controls indicating that there may be a mechanism by which infection leads to diminished E2-2 expression [19,27]. To address if freshly-isolated human pDCs modify E2-2 expression after stimulation, we began by identifying peripheral pDCs that expressed E2-2. Flow cytometric analysis of E2-2 in freshly isolated primary human PBMCs verified that high E2-2 expression is restricted to pDCs (Figure 1A). Recent analysis of DC populations has revealed a subset of mDCs expressing CD123 that may fall into traditional CD123+ BDCA2+ pDC gates [8,14,34]. These AXL+ Siglec 6+ mDCs produce less IFN than pDCs and can produce IL-12; they are also more efficient at stimulating T cell proliferation than traditional pDCs [14]. Since AXL+ Siglec6+ mDCs express pDC markers, it is possible that some of the attributes previously assigned to pDCs actually belong to AXL+ Siglec 6+ mDCs, particularly the capacity to present antigen [2,14]. To prevent contamination of our pDC populations with AXL+ Siglec 6+ DCs we used CD11c to exclude mDC populations and monocytes (Figure 1A). Additionally, we determined that negative-selection of pDCs by magnetic activated cell sorting removed all AXL+ Siglec 6+ cells from the cell culture (Figure 1B). CD11c+ cells expressed a low level of E2-2 and CD3+ T cells were E2-2 negative, in accordance with previous literature (Figure 1C). To investigate if stimulation of pDCs modulates E2-2 expression, PBMCs were treated with the TLR7 ligand R848 for 6 h. Maximal IFN production in response to R848 occurs at 2 h [35], however, by this time there was no significant alterations in E2-2 protein levels. Continued stimulation of PBMCs lead to a significant decrease in E2-2 expression at 6 h (Figure 1D). We confirmed that diminished protein levels of E2-2 also corresponded with significantly downregulated mRNA expression (Figure 1E). mRNA levels were significantly lower by 2 h in the R848 treated samples indicated that E2-2 mRNA production is inhibited prior to a significant drop off in protein levels. This provides evidence that E2-2 expression can be altered during maturation of primary human pDCs.

### 3.2. Differential Expression of E2-2 is Associated with Functional and Phenotypic Differences

**Figure 2:**
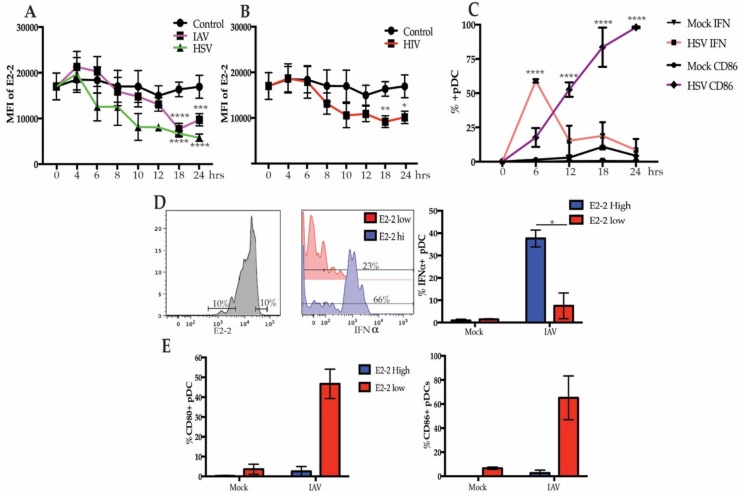
E2-2 expression after viral stimulation is associated with functional and phenotype differences. (**A**,**B**) Expression of E2-2 in pDCs after PBMCs were stimulated with herpes simplex virus type 1 (HSV) or influenza A virus (IAV) at a multiplicity of infection (MOI) of 1 (**A**) or 500 ng p24 equivalents/mL of HIV-MN (**B**); *n* = 5 independent experiments. (**C**) pDCs stimulated with HSV for 0–24 h were measured for intracellular IFNα and CD86 expression by flow cytometry, *n* = 3 independent experiments. (**D**,**E**) Gating strategy to compare the 10% highest and lowest E2-2 expressing pDCs after stimulation with IAV for 24 h. Comparison of the percent IFNα+ cells (**D**) and CD80+ and CD86+ (**E**). (**D**,**E**), *n* = 4 independent experiments. Data are presented as means ± SEM. *p* values < 0.05 were considered significant. * *p* < 0.05, ** *p* < 0.01, *** *p* < 0.001, **** *p* < 0.0001.

PBMCs treated with influenza A virus (IAV) and herpes simplex type 1 (HSV), which signal through TLR7 and TLR9, respectively, demonstrated a similar pattern of downregulation of E2-2, in which E2-2 was diminished after peak IFN response. By 12 h, E2-2 expression in the pDC population was not significantly decreased in response to either IAV or HSV-1. However, at 18 h, E2-2 was significantly lower and stayed suppressed at 24 h (Figure 2A). Though there was a slight upward trend of E2-2 expression at 24 h during stimulation with IAV, there was not a significant increase in E2-2 expression. As with IAV, pDCs also respond to HIV-1 via TLR7 signaling [36] and HIV-1 stimulation also caused significant downregulation of E2-2 (Figure 2B). Optimal intracellular IFNα expression in response to viral stimulation in pDCs is seen 6–8 h [37,38] and upregulation of costimulatory markers occurs after IFNα production subsides. We verified this by stimulating PBMCs with IAV for 24 h and measured IFNα and CD86, a costimulatory molecule, expression every 6 h. The percent of IFNα producing cells peaked at 6 h and rapidly declined over the next 18 h (Figure 2C). Conversely, CD86 was not significantly upregulated until 12 h (Figure 2C). These data support that downregulation of E2-2 occurs after pDCs have diminished production of IFNα and begun to upregulate costimulatory molecules required for T cell activation. 

In murine pDCs and human pDC cell lines, diminished E2-2 expression causes changes in phenotype and function marked by decreased IFNα expression and increased costimulatory markers [15]. Since pDC cell lines do not produce an equivalent concentration of IFNα compared to primary human pDCs and do not fully replicate primary pDC function [21,22,23], we interrogated these changes in our system. We stimulated PBMC with IAV for 24 h, then stained and gated on the 10% of pDCs with the highest and lowest E2-2 expression and evaluated their intracellular IFNα production (Figure 2D). After stimulation with IAV, a greater percentage of E2-2 high pDCs were IFNα+ compared to E2-2 low pDCs (Figure 2D). In contrast, E2-2 low pDCs upregulated the costimulatory markers CD80 and CD86 by 24 h, while E2-2 high pDCs did not (Figure 2E). Together, these data suggest that E2-2 may be regulated in response to viral activation, and changes in E2-2 expression are associated with reduced IFNα production and upregulation of costimulatory markers.

### 3.3. Decreased E2-2 Expression Is Not Induced by IFNα Receptor Signaling

**Figure 3:**
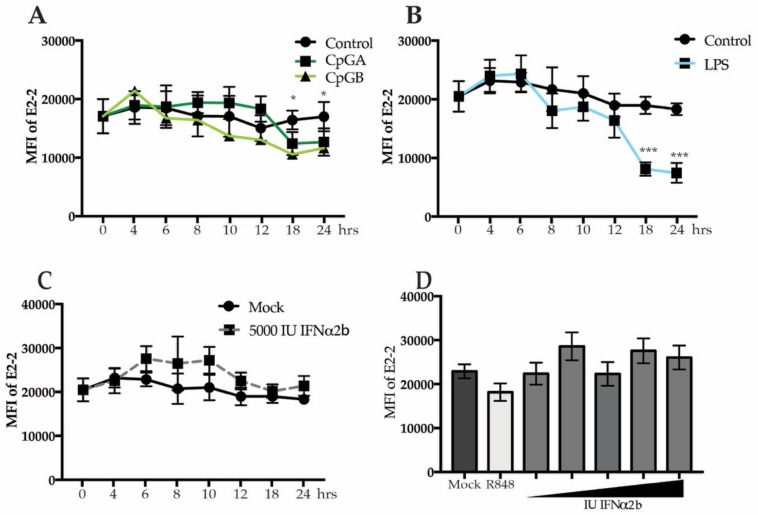
IFNα receptor signaling does not diminish E2-2 expression. PBMCs were treated with 5 ug/mL of CpGA or CpGB (**A**), 200 ng/mL lipopolysaccharide (LPS), (**B**) or 5000 IU recombinant IFNα2b (**C**) for up to 24 h. (**D**) PBMCs were treated with 10, 100, 1000, 5000, or 10,000 IU of IFNα2b for 24 h. *n* = 5 independent experiments. Data are presented as means ± SEM. *p* values < 0.05 were considered significant. * *p* < 0.05, ** *p* < 0.01, *** *p* < 0.001.

Production of IFNα by pDCs can result in an IFNα receptor (IFNAR)-mediated positive feedback loop [39]. To address the role of IFNAR signaling in E2-2 regulation, we utilized the differential effects of oligodeoxynucleotides CpGA and CpGB on pDCs. In human pDCs, CpGA induces robust IFN production leading to an autocrine IFN signaling loop. Conversely, CpGB induces a very low level of IFN production which is below the threshold required for autocrine signaling [39]. After 18 h, PBMCs treated with either CpGA or CpGB decreased E2-2 (Figure 3A), arguing against an autocrine IFNα signaling loop for E2-2 downregulation.

We previously reported that lipopolysaccharide (LPS) activates pDCs via TLR4 resulting in enhanced TLR4 and IRF7 expression via an NFκB-dependent pathway but with no IFNα production [7]. To determine if TLR4 signaling results in altered expression of E2-2 in the absence of IFNα production, we stimulated PBMCs with LPS and then measured E2-2 expression. By 18 h, there was a significant reduction in E2-2 in LPS-treated PBMC (Figure 3B) that followed the same kinetics of E2-2 downregulation as cells stimulated with virus. To confirm that IFNα signaling does not play a role in E2-2 down-regulation in pDC, we treated PBMCs with recombinant IFNα2b for up to 24 h. There was no decrease in E2-2 expression at any of the time points, and, in fact, there was a small but not significant increase in E2-2 in the treated samples (Figure 3C). Furthermore, increasing concentrations of IFNα did not induce a change in E2-2 expression (Figure 3D). These data indicate that IFNAR signaling does not cause attenuated expression of E2-2.

### 3.4. Secreted Cytokines from Neighboring Cells Lead to Downregulation of E2-2

**Figure 4:**
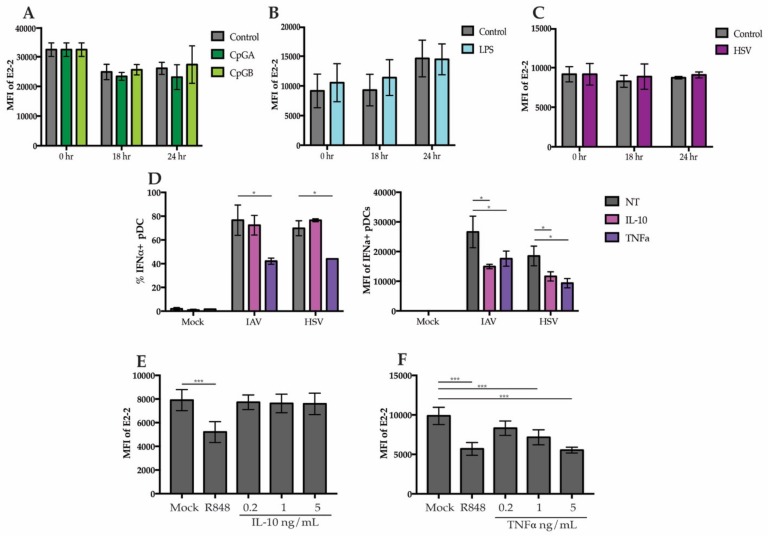
E2-2 expression is mediated by a pDC extrinsic signal (**A**–**C**) pDCs enriched to a purity >90% were stimulated for 18 or 24 h with (**A**) 5 ug/mL CpGA or CpGB (**B**), 200 ng/mL LPS or (**C**), HSV. (**D**) PBMCs were cultured with 5 ng/mL of IL-10 or TNFα and stimulated with HSV or IAV. Intracellular IFNα production was measured at 6 h. (**E**,**F**) PBMCs were treated with either 10 µM R848, 0.2, 1, or 5 ng/mL IL-10 (**E**) or TNFα for 6 h then E2-2 expression was measured by flow cytometry. *n* = 3 independent experiments. Data are presented as means ± SEM. *p* values < 0.05 were considered significant. * *p* < 0.05, ** *p* < 0.01, *** *p* < 0.001.

To determine if downregulation of E2-2 was due to direct sensing of TLR agonists by pDCs or in response to signals secreted by neighboring cells, we stimulated purified pDCs with TLR7, TLR9, and TLR4 agonists. pDCs were enriched to >90% purity using negative selection and stimulated for 24 h. After stimulation, there were no significant changes in E2-2 expression with HSV, CpGA, CpGB, or LPS in the purified pDCs (Figure 4A–C). Since we did not observe changes in purified pDCs, this suggests that the altered expression of E2-2 in PBMC cultures is not a direct effect. Other cells, such as monocytes and mDCs also produce a variety of cytokines in response to LPS and viruses [40] and it has previously been demonstrated that certain cytokines, including TNFα and IL-10 can alter pDC function [41,42,43]. Indeed, adding TNFα to the media before stimulation with HSV and IAV significantly decreased the total percent of IFNα+ pDCs and the average amount of IFN produced by each cell, as determined by the intracellular mean fluorescent intensity (Figure 4D). The addition of IL-10 did not decrease the percent of IFNα+ pDCs but did significantly diminish the mean fluorescent intensity (Figure 4D).

To determine whether the IL-10 and TNFα may be responsible for regulation of E2-2, we treated PBMCs with TNFα and IL-10. Treatment with IL-10 did not cause a decrease in E2-2 (Figure 4E). However, TNFα treatment alone was sufficient to decrease E2-2 expression by 6 h (Figure 4F). This reveals that a potential mechanism for TNF regulation of pDCs is through downregulation E2-2. resulting in Inhibition IFN Production.

### 3.5. Regulation of E2-2 Is Mediated by TNFα Produced by Monocytes

**Figure 5:**
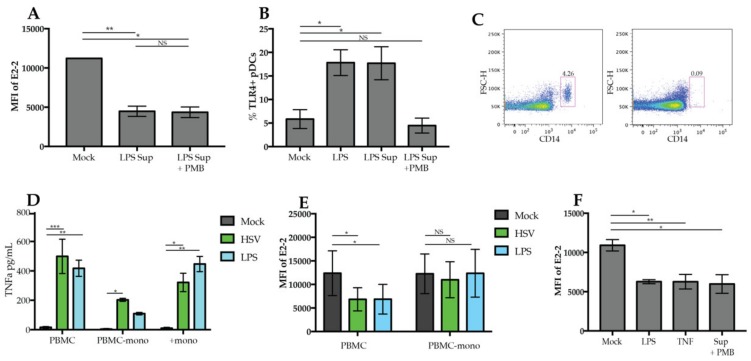
Monocyte-derived TNFα drives downregulation of E2-2. (**A**) Enriched pDCs were stimulated with 1 mL of supernatant from PBMCs stimulated with 200 ng/mL LPS for 24 h with or without the addition of polymyxin B (PMB) 30 min before the addition of LPS. (**B**) PBMCs were stimulated for 6 h with LPS, supernatant from PBMC stimulated with LPS or supernatant plus PMB, then TLR4 expression was quantified by flow cytometry (**C**) Monocytes were depleted from freshly isolated PBMCs. Depletion was confirmed via flow cytometry as indicated in the representative dot plot. (**D**) Supernatant collected from PBMCs, PBMCs depleted of monocytes (PBMC-mono), and positively-selected monocytes (+mono) stimulated with LPS for 24 h were measured for presence of TNF α by ELISA. (**E**) PBMCs or PBMC-mono cultures were cultured with either HSV or LPS for 24 h then E2-2 expression was measured. (**F**) 1 mL of supernatant from LPS-stimulated positively- selected monocyte cultures was used to culture PBMCs for 24 h. Supernatant was pretreated with PMB for 30 min prior to culture to prevent the activation of PBMCs with LPS. *n* = 3 independent experiments. Data are presented as means ± SEM. *p* values < 0.05 were considered significant. * *p* < 0.05, ** *p* < 0.01, *** *p* < 0.001, **** *p* < 0.0001.

To determine if cytokines present in the supernatants of TLR-activated PBMCs are sufficient to induce downregulation of E2-2 in pDCs during stimulation, we cultured enriched pDCs with supernatants collected from PBMCs stimulated with LPS. These supernatants resulted in the downregulation of E2-2 (Figure 5A). To rule out the possibility that LPS in the supernatants was responsible for the downregulation of E2-2, supernatants were treated with polymyxin B, which prevents LPS binding to TLR4 and the activation of pDCs [7]. Downregulation of E2-2 did occur when supernatants were pretreated with polymyxin B (Figure 5B). To ensure that polymyxin B prevented LPS activating pDCs directly, we measured upregulation of TLR4 on pDCs. Stimulation with LPS and supernatant caused a significant increase in the percent of pDCs expressing TLR4. As we have previously reported [7], treatment with polymyxin B prevented upregulation of TLR4 (Figure 4F). These results indicate that E2-2 downregulation is not specific to TLR stimulation, but rather a response to the milieu of cytokines present in the media.

Monocytes are a major source of TNFα, so to determine if they contribute to the regulation of E2-2, we depleted monocytes from PBMCs (PBMC-mono) (Figure 5C) and stimulated with HSV and LPS. In response to HSV, PBMC-mono cultures produced a significant amount of TNF relative to mock, however, it was drastically reduced compared to whole PBMC cultures (Figure 5D). Our lab and others have reported that pDCs produce TNFα in response to viruses [44,45]; therefore, the TNFα production in PBMC-mono cultures may be a result of TNFα production by pDCs. Positively-selected monocytes cultured alone also produced a significant amount of TNFα after stimulation with HSV or LPS (Figure 5D) indicating that monocytes are a significant source of TNFα in PBMCs. When we stimulated PBMCs without the presence of monocytes, we found that there was no E2-2 downregulation in response to LPS or HSV (Figure 5E). Stimulation of PBMCs with supernatant collected from positively-selected monocytes did induce a significant reduction of E2-2 expression (Figure 5F). Therefore, these data support that the E2-2 downregulation we observed is due to monocytes producing TNFα that then triggers pDCs to alter E2-2 expression. 

## 4. Discussion

Regulation of pDCs and their robust production of type I IFNs is pivotal in preventing aberrant inflammation [29,30,46]. While the production of type 1 IFNs is essential for both innate and adaptive immune responses, uncontrolled production can contribute to inflammatory disorders [46,47]. E2-2 is a master regulator of pDC function, promoting and repressing genes required for development of pDCs [9]. Since E2-2 expression is required for pDC maintenance, alterations in E2-2 can lead to a cell drastically changed in phenotype and function. In mice, inducible knockout of E2-2 gives rise to a cell that is markedly decreased in its ability to produce IFNs but more adapted to stimulate T cell responses [15]. This is recapitulated in human pDC cell lines where E2-2 knockdown decreases IFN production and increases the ability to induce T cell proliferation [15,17], though pDC leukemic cells lines produce little or no IFN and do not fully represent primary human pDC function [20,21,22,23]. It has also been demonstrated that mouse and human pDCs downregulate E2-2 in response to chronic viral infection [16,19]. However, the signals leading to E2-2 modulation have not been elucidated.

High E2-2 expression is restricted to pDCs, with nominal expression in other DC subsets. During development, pDCs upregulate E2-2 while in the bone marrow before egressing into the blood [2]. We confirmed that primary human blood pDCs have much higher expression of E2-2 compared to other immune cells including mDCs and T cells. We also demonstrated in pDCs, peak intracellular IFNα occurs 6 h after the start of HSV and IAV stimulation and quickly diminishes [38]. Conversely, costimulatory markers are not significantly upregulated until 12 h and continue to increase in expression over time. We found that when stimulated, pDCs downregulated E2-2 during the process of costimulatory marker upregulation. During the time that E2-2 tapered, pDCs upregulated CD80 and CD86 and began to cease IFN production. These kinetics suggest that E2-2 may have a role in the upregulation of costimulatory markers during activation and in the moderation of IFN production.

Furthermore, we found that pDCs with lower E2-2 expression are more likely to have upregulated CD80 and CD86 compared to pDCs with high E2-2 expression and that the percent of pDCs producing IFNα was significantly greater in pDCs with high E2-2. These changes in function may represent the coordinated shift of pDCs from an interferon producing cell to antigen presenting cell. This transition is important to prevent overproduction of proinflammatory cytokines and for the activation of the adaptive immune system and may indicate that purified pDC populations are capable of stimulating T cells after they have downregulated E2-2.

IFNα receptor signaling in primary human pDCs results in a positive feedback loop and blocking the IFNα receptor decreases overall IFN production [39]. CpGB does not trigger a strong enough IFN response to lead to an IFNα feedback loop, however pDCs showed a similar extent of E2-2 downregulation in response to both CpGA and CpGB. Directly exposing cells to exogenous IFNα did not attenuate E2-2 protein expression, even at IFN concentrations vastly greater than physiological levels. There was even a small but not significant increase in E2-2 expression after exposure to IFNα. Rather than suppressing, IFN may, instead, support continued expression of E2-2. Since viruses, CpGA, and CpGB caused diminished E2-2 expression, it does not appear that IFNα receptor signaling has a fundamental role in E2-2 downregulation during pDC maturation.

In addition to TLR7 and TLR9, human pDCs also express TLR 4 [7,48]. Previous reports from our lab found that LPS activation of pDCs induces upregulation of IRF7 and TLR4. Stimulating pDCs with LPS prior to viral stimulus leads to a more robust IFN response which is mediated by the increased IRF7 expression [7]. This response led us to investigate if E2-2 expression has a role in pDCs response to LPS. We found that in PBMC cultures, E2-2 expression was repressed after stimulation with LPS. Following the pattern after viral stimulation, E2-2 was significantly decreased by 18 h.

When we attempted to replicate our results from PBMCs in enriched pDC populations, we were unable to detect any alterations in E2-2 expression. It has been shown by our lab and others that pDC function can be altered by cytokines from neighboring cells [40,41,49]. TNFα and IL-10 have both been reported to inhibit IFNα production in pDCs, however the mechanism leading to this inhibition is unclear [41,49]. Indeed, when we pretreated with IL-10 there was diminished mean fluorescent intensity of IFNα after viral stimulation. This indicates that, although the percent of pDCs producing IFN remained the same, the amount of IFN produced on a per cell basis was attenuated. Adding TNFα to the media significantly inhibited both the percent IFNα+ pDCs and the mean fluorescent intensity of IFNα. To determine if IL-10 or TNFα alter E2-2 expression, we treated PBMCs with both cytokines and found that only TNFα was sufficient to induce downregulation of E2-2. Alteration of E2-2 expression in response to TNFα may represent a mechanism for the previously observed attenuation of pDC activation in response to TNFα [41].

To determine if there was a signal in the supernatant of PBMCs that caused downregulation of E2-2, we collected media from PBMCs stimulated with LPS and used this media to culture autologous pDCs. This resulted in significantly decreased E2-2. To ensure that LPS was not triggering signaling pDC through TLR4, we added PMB to the supernatant. PMB prevents binding of LPS to TLR4 and inhibits activation of pDCs with LPS [7]. In accordance with our previous observations, PMB prevented the upregulation of TLR4 in LPS treated pDCs. Taken together, these results show that the downregulation of E2-2 is not a direct effect but rather an indirect effect mediated by other cells in the PBMC population. Similar mediation of pDC activation and IFN production by TNFα has been described [40,41], but the molecular basis has not been well-defined.

Although PBMCs are a very heterogenous population comprised of many immune cell populations, we investigated the potential role of monocytes in E2-2 regulation because monocytes have been implicated in the regulation of pDCs [40,41,49]. Depleting monocytes from PBMCs diminished the concentration of TNFα in the supernatants; however, there was still a low level of TNFα present. This TNFα may come from pDCs, which are known to produce TNFα in response to viral stimuli [44]. The concentration of TNFα in the monocyte depleted cultures was approximately half that of the PBMC cultures. This low concentration may not be enough to signal pDCs to downregulate E2-2. When PBMCs were stimulated in a monocyte-depleted PBMC culture, there was no altered expression of E2-2 in response to either LPS or HSV. Supernatants collected from purified monocyte cultures stimulated with LPS and HSV also induced downregulation of E2-2 in pDC. Together, these data show that TNFα produced by monocytes has a direct effect on the expression of E2-2 in pDCs.

Given that diminished E2-2 results in a greater capacity to stimulate T cells, altered E2-2 expression may assist in the development of a proficient antigen presenting pDC. The concept that pDCs shift from interferon producing cells to antigen presenting cells was suggested early in pDC research [50,51,52,53,54]. In 2003, the first direct evidence that pDCs present antigen and stimulate T cells was reported when Fonteneau et al. showed that pDCs exposed to IAV efficiently stimulated T cell proliferation, later reports also colloborate that pDCs can present antigen [52,55]. However, it has recently been debated if antigen presenting pDCs are instead an entirely distinct subset of AXL+ Siglec6+ mDCs [14,34]. Analysis of single-cell RNA sequencing and mass spectrometry of human PBMCs revealed these mDCs demonstrate characteristics of both pDCs and mDCs with low expression of E2-2 [8,14,34]. We have shown that pDCs reduce E2-2 expression in response to stimulation with TNFα; therefore, it is possible that some E2-2 low mDCs are pDCs that have encountered local or circulating TNFα and correspondingly downregulated E2-2.

There are several reported circumstances in which pDC function is altered or inhibited in response to infection, cytokines, or cross-linking of surface markers. For example, pDCs are well known to be adversely affected by chronic HIV infection [28,31,32], and our previous work has shown that E2-2 expression is decreased in HIV-infected individuals [27]. It is possible that the loss-of-function observed in pDCs from people living with HIV may be caused by altered E2-2 expression. There are also other mechanisms that cause inhibition of pDC function such as cross-linking of BDCA2, CD123, and ILT7 [56,57,58,59,60,61]. However, if E2-2 has a role in inhibition in these contexts has not been confirmed. It would be intriguing to investigate if the attenuation observed after cross-linking or receptor binding is also mediated by E2-2. Conversely, it is plausible that pDCs from patients with interferonopathies may have deviant upregulation of E2-2 expression. By fully understanding the signals that regulate E2-2 and pDCs, we may be able to rescue function during chronic HIV infection and prevent over-activation of pDCs in autoimmune disorders.

In conclusion, we have shown that pDCs alter E2-2 expression in response to monocyte-derived TNFα. We propose a mechanism in which pDCs with high E2-2 expression initially produce IFN, but as monocytes begin to secrete TNF, pDCs downregulate E2-2. Attenuated E2-2 expression leads to decreased IFN production and upregulation of costimulatory markers. Ultimately, this contributes to a shift from a cell adept at IFN production to a cell equipped to present antigen, bridging the innate and adaptive compartments of the immune system.
